# Generation and Tuning of Semiconductor Electronic and Functional Properties through Electrochemical Patterning

**DOI:** 10.1021/accountsmr.5c00104

**Published:** 2025-08-01

**Authors:** Denis Gentili, Edoardo Chini, Massimiliano Cavallini

**Affiliations:** 9327 Istituto per lo Studio dei Materiali Nanostrutturati (ISMN)-Consiglio Nazionale delle Ricerche (CNR) Via P. Gobetti 101, 40129 Bologna, Italy

## Abstract

This Account presents surface electrochemical nanopatterning as a powerful and underexplored strategy for engineering the electronic and functional properties of electrochemically active materials. By enabling precise, localized manipulation of electronic states at the micro- and nanoscale, this technique offers a unique pathway to unlock and control intrinsic material properties. These capabilities open new frontiers in materials science, with implications ranging from catalysis to the fabrication of advanced, multifunctional devices.

Traditional lithographic techniques, such as photolithography, electron beam lithography, and nanoimprinting, mainly focus on shaping surface topography. In contrast, electrochemical nanopatterning introduces a fundamentally different approach: it modifies the material itself. By changing oxidation states, creating or healing defects, and tuning surface chemistry, this method allows for direct control of material properties. Consequently, it greatly expands the range of applications, enabling the development of materials with customized electronic and functional features.

This Account focuses specifically on stamp-assisted electrochemical lithography (ECL), a versatile and scalable technique. We start by outlining the fundamental principles of ECL, including the electrochemical processes that drive it, namely oxidation, reduction, and defect generation. Next, we trace its historical development and highlight its advantages over traditional nanofabrication methods, particularly in terms of simplicity, cost-effectiveness, and compatibility with a wide range of materials. Through a curated selection of case studies, we demonstrate how ECL can be used to (i) generate and tune electronic properties, (ii) impart various functional behaviors, and (iii) achieve spatially controlled defect engineering, especially in semiconductors.

Crucially, the ability to fabricate large-area samples has allowed us to harness size-dependent properties that were previously inaccessible in electrochemical nanolithography performed via scanning probe techniques, such e catalysis and the in situ fabrication of nanoclusters. These findings dramatically expand the scientific and technological potential of ECL, opening new avenues for innovation and application. The example cases were selected for their relevance to current challenges in materials science and emerging technologies. Notable applications include in situ healing in resistive switching devices, the development of critical-element-free catalysts, and the direct fabrication of active components within devices. Many of these studies were pioneering at the time of publication and have only recently gained broader recognition due to the growing interest in sustainable, low-cost, and scalable nanofabrication techniques.

We emphasize ECL’s unique capabilities in enabling regenerable resistive switching, spatially selective nanoembedding of functional nanoparticles, and creating functional surface patterns. These features position ECL as a promising tool for bridging the gap between fundamental research and practical device integration. Moreover, the method’s compatibility with ambient conditions and its potential for large-area processing make it particularly attractive for industrial applications.

In the final section, we discuss the frontier and the perspectives of ECL. We propose strategies to enhance resolution, reproducibility, and integration with existing manufacturing platforms. We also outline future directions, including the development of hybrid patterning approaches. Looking ahead, we envision ECL playing a central role in the development of next-generation materials and devices, particularly in fields where precise control over local properties is essential for both performance and functionality.

## Graphical abstract

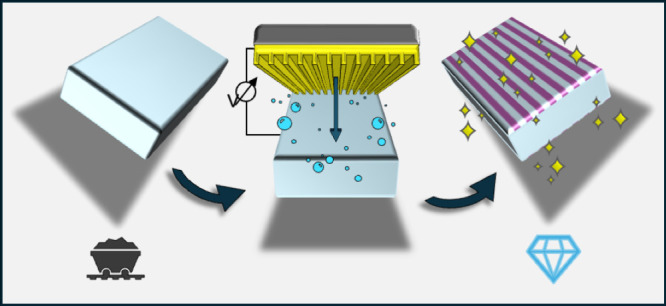

## 1 Introduction

The precise control of the spatial arrangement of materials on surfaces, known as surface patterning, provides an extraordinary variety of opportunities in material science and technology.^1,2^ Within the micro and nanoscale range, patterning has proven to be an efficient way, capable of enhancing existing physical properties and generating entirely novel properties;^3,4^ moreover, it allows for the precise positioning of nano-objects in controlled locations within devices, which is a critical issue in technology.^5^ Among the numerous techniques developed for nanopatterning, electrochemical methods play a pivotal role due to their simplicity and versatility.^2,6,7^ Unlike other techniques such as photolithography,^8^ electron beam lithography^9^ and nanoimprinting,^10^ electrochemical techniques allow the controlled modification of material properties by altering oxidation states, introducing defects, and changing surface chemistry.^11,12^ These properties enormously expand the potential applications of materials by generating and tuning new properties. Principal applications include nanofabrication,^2^ surface functionalization,^11^ and, more recently, defect engineering,^13,14^ among others. Over the past two decades, numerous studies have demonstrated the potential of electrochemical patterning techniques, usually using scanning probe lithography (SPL).^6,7,15^ SPL represents an advanced suite of nanolithographic methods that utilize scanning probes, with local anodic oxidation nanolithography emerging as the most used technique.

Despite its extraordinary potential for innovative nanoscale applications, SPL faces two critical limitations. i- The SPL techniques are inherently serial, meaning they fabricate nanostructures sequentially, one at a time. ii- They are restricted by the scanning range of the piezoelectric system, resulting in lower throughput when producing large quantities of nanostructures. Although recent developments in the instrumentation have led to a significant increase in the speed of fabrication through parallelization^16^ and automation,^17^ these constraints still significantly impact the broader technological influence that SPL could have in the field of nanotechnology, particularly regarding patternable areas.^6^ One of the main strategies for overcoming the limitations of electrochemical-based SPL is stamp-assisted electrochemical lithography (ECL), which uses a conductive stamp with arrays of protrusions that replace the SPM probe.^18^ ECL significantly improves the throughput of the process, allowing the patterned areas to expand from micrometres to centimeters scale.^19,20^ Table 1 compares the key characteristics, such as throughput, resolution and drawbacks of parallel ECL with the most widely used techniques for nanofabrication and patterning.

**Table 1:** Comparison of the Principal Characteristics of the Most Widely Used Techniques for Patterning

**Method**	**Principle**	**Advantages**	**Drawbacks**
Photolithography^21^	Photomask fabrication	-Highly consolidated.	-Optically limited resolution.
		-Large area.	-Successive steps required
Nanoimprinting^22^	Morphological Replication of nanoscale patterns	-High resolution	-No chemical modification.
		-Large area.	-Limited to soft materials.
Soft lithography	Transferring material from flexible stamps to a substrate	-Versatility.	-Poorly applicable to inorganic materials.
		-Simplicity.	
Local thermal treatments (Laser irradiation and scanning thermal microscopy^23,24^	Exploit thermal effects by locally heating.	-Simplicity	-Limited control of the effects
Electron-beams lithography.^25^	Controlled electron irradiation	-High resolution	-Requires hi-tech equipment.
			-No organic substrates
Single ion implantations.	Addition of dopants on a material in controlled positions by irradiation.	-High resolution.	-Requires hi-tech equipment.
		-Compositional control	-Uncontrolled local alterations of substrate
Scanning Probe Lithography.^26,27,28^	Exploit mechanical, and electrochemical probe-surfaces interaction s	-Versatility.	-Requires hi-tech equipment.
		-Simplicity.	-Low throughput.
		-High resolution	Patternable area ≪1 × 1 μm^2^.
Electrochemical nanolithography:	Local electrochemical reaction	-Large area.	-Applicable on conductive substrates.
		-Tailoring of properties	

This Account aims to raise awareness within the materials science community about the capabilities of stamp-assisted ECL. Our selection of practical examples was guided by their relevance to current challenges in materials science and technology, including in situ healing in resistive switching, the development of new catalysts free from critical elements, and the in situ fabrication of active components in devices, which are currently hot topics addressable by ECL. Many of the studies we included were pioneering at the time of their publication and have only recently gained wider recognition due to the increasing interest in these areas.

By manipulating materials at the submicron and nanoscale, ECL unlocks innovative pathways for controlling material properties, offering vast opportunities for progress by extending the benefits of local electrochemistry to large-area samples. The ability to fabricate large-area samples has allowed us to harness size-dependent properties that were previously inaccessible in electrochemical nanolithography performed via scanning probe techniques. These include, but are not limited to, catalysis and the in situ formation of nanoclusters. These findings dramatically expand the scientific and technological potential of ECL, opening new avenues for innovation and application.

We discuss the fundamental principles, historical development, and the electrochemical processes exploited in ECL, such as oxidation, reduction, and defect creation, and introduce the key applications of ECL, including the customization of electronic and catalytic properties, the development of regenerable and *in situ* healing resistive switches, the spatially controlled nanoembedding of functional nanoparticles, and the enabling of functional patterns. Finally, we present perspectives and strategies to advance ECL from the nanoscale to the atomic scale, highlighting open challenges and future directions.

## 2 Origin and Development of Electrochemical Nanolithography

Since the advent of Scanning Probe Microscopy (SPM), one of its most widespread applications has been the development of SPL, nanolithographic techniques that use the probe to modify surfaces through mechanical and/or electrical interactions.^6,7^ Thanks to the probe’s nanometric size, SPL can achieve remarkable spatial resolution under 10 nm with standard instrumentation.^6,29^ However, the low throughput and the requirement for advanced SPM instrumentation pose significant limitations for technological applications that demand high throughput.

Among the SPL techniques, Local Anodic Oxidation (LAO) is the most widely used. LAO exploits local electrochemical oxidation to chemically or morphologically modify a conductive surface; predominantly, it has been used for silicon oxidation.^6^ In LAO, a conductive AFM tip is positioned near an electroactive substrate in a controlled high-humidity environment. In these conditions, a water meniscus develops between the probe and the surface through capillary forces. When a voltage is applied between the probe and the substrate (typically <1.5 V), an electrochemical reaction occurs. A positive bias induces local oxidation on the surface. The reaction is spatially confined within the meniscus, allowing for precise pattern formation limited by the size of the probe. The duration, type (DC or AC), and the value of the bias determine the nature of the reaction. Moving the AFM tip during the process generates the desired pattern. Figure 1a shows a schematic representation of LAO.

**Figure 1:**
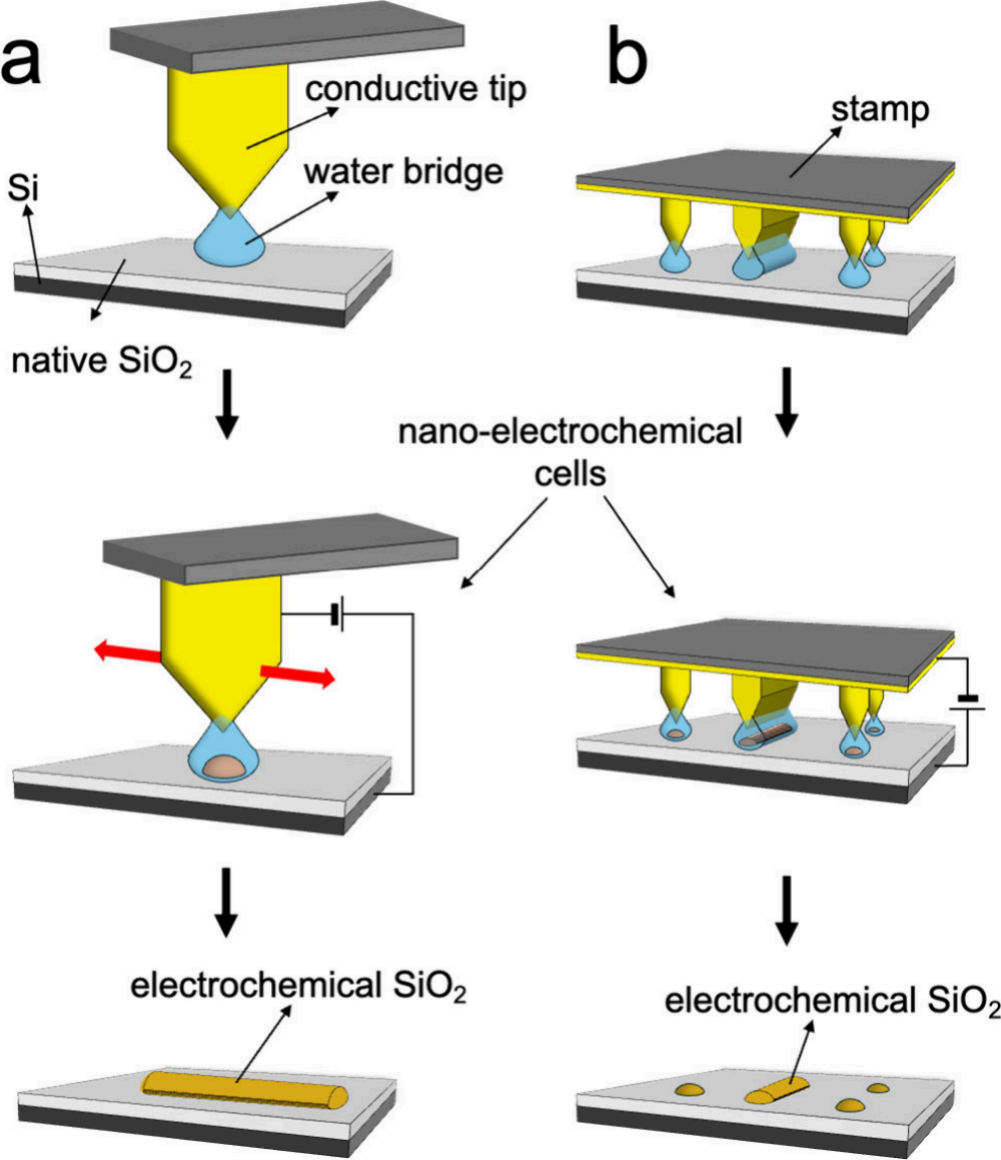
Schematic representation of the electrochemical lithography. (a) An AFM probe performs local anodic oxidation. (b) Parallel ECL process.

ECL evolved from SPL by utilizing a stamp with a prefabricated array of protrusions instead of an SPM probe, thereby overcoming the throughput limitations. The concept of parallel oxidation using a stamp was introduced in 2000 by Mühl et al.^18^ Then in 2003, several groups demonstrated independently parallel ECL: Hoeppener et al. demonstrated parallel electrochemical patterning through constructive lithography, utilizing a metallic grid as a stamp;^11^ Yokoo introduced parallel silicon oxidation naming the process “Nanoelectrode Lithography”;^30^ eventually, our group, in collaboration with Garcia’s group at CSIC, systematically developed stamp-assisted ECL in 2003, enabling the fabrication of logic patterns on silicon surfaces.^19^ ECL combines the SPM-based LAO with lithographically controlled wetting,^31^ an unconventional lithographic technique that precisely manages the placement of a liquid or solution beneath the protrusions of a stamp positioned near a surface used for nanopatterning of various soluble materials.^5,32,33^

In ECL (Figure 1b), a polymeric stamp—typically made of metallized elastomer—is placed on the substrate in a high-humidity environment (RH > 95%). In these conditions, several water menisci develop between each stamp motif and the substrate, driven by capillary action, leading to forming an array of 2-electrode nanoelectrochemical cells.

Applying bias to the substrate induces a localized electrochemical reaction, confined beneath the stamp motif, allowing precise pattern formation that replicates the stamp features. The size and shape of the stamp motifs determine the spatial resolution and the shape of the printed structures. Typically, ECL requires a bias 1 order of magnitude higher (>10 V) than in LAO.

The stamps are fabricated by replica molding from a master, followed by metallization (usually Au), to ensure electrical conductivity. The master mold is produced using conventional lithography methods.^8,9^ Importantly, in ECL the printed features are often smaller than the physical dimensions of the stamp features, because of the tip-effect at the apex of the stamp features, which confines the water meniscus and enhances the electric field (Figure 1b).^19,20^

ECL exhibits lower spatial resolution (i.e., the printed feature size), compared to LAO (ECL < 90 nm over areas of 1 × 1 cm^2^, LAO < 10 nm over areas of 100 × 100 nm^2^, respectively). This limitation is due to the larger size of the stamp, in comparison to the SPM tip, and the lower control of operative conditions with respect to LAO (In ECL, the same operative conditions are applied for all features, whereas in LAO, the conditions are optimized for each structure). On the other hand, ECL enables the creation of thicker structures, exceeding 20 nm, while LAO is limited to less than 2 nm. This thickness can be adjusted by acting on bias voltage, time, stamp-surface distance and relative humidity. The process can be repeated in the same areas to create complex structures, to increase the effect of the process and to combine different electrochemical processes in specific zones. Stamp alignment and positioning are achieved using microtranslators beneath the sample holder and by stamp rotation.^20^

ECL was initially used for silicon oxidation to demonstrate the parallelization capabilities of scanning probe lithography techniques.^18–20,30^ However, the ability to produce large-area samples has greatly expanded the scope of ECL beyond basic nanofabrication. This development has allowed precise control over local surface roughness,^34^ the engineering of patterned^20^ structures to modify wetting properties,^3^ and template growth.^35^

The fabrication of large, well-defined patterned areas that are compatible with standard characterization techniques has enabled the observation of intriguing electrochemical phenomena, such as the formation of nanoclusters and changes in surface wettability. These findings have significantly expanded the scope of ECL, opening the way for novel technological applications. These include, but are not limited to, the following:

### 2.1 In-Situ Synthesis and Patterning of Silicon Nanoclusters (Ref 34)

Alternating current ECL (AC-ECL) was used to synthesize *in situ* silicon nanoclusters (Si NCs) embedded within a SiO_2_ matrix. The formation process involves the oxidation of silicon substrates under a positive bias, producing metastable species such as partially oxidized silicon (Si­(I) and Si­(III)).^36^ Upon applying a negative bias, these species undergo electrochemical reduction, leading to the formation of Si NCs within the oxide matrix. The positioning of these NCs is controlled by the distribution of the stamp features. Importantly, this phenomenon is only observable in large-area samples because Raman spectroscopy was used to demonstrate the NCs formation (Figure 2a).

**Figure 2:**
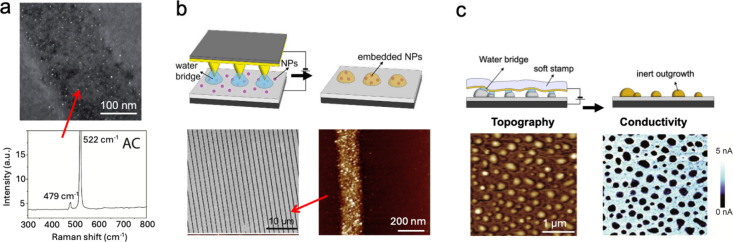
(a) Top: SEM image of stripes printed in AC mode on Silicon surface showing the formation of Si nanoclusters inside SiO_2_. Bottom: Corresponding Raman spectra showing a peak at 470 cm^–1^ related to the formation of Si nanoclusters. Reproduced with permission from ref 34. Copyright 2019 The Royal Society of Chemistry. (b) Top: Scheme of nanoembedding. Bottom: SEM and AFM image of NPs embedded in SiO_2_ (Z scale 0–20 nm). Reproduced with permission from ref 37. Copyright 2010 The Royal Society of Chemistry. (c) Top: Scheme of electrochemical decomposition of outgrowths. Bottom: Topography (Z scale 0–20 nm) and the corresponding AFM conductivity map recorded following the local electrochemical reduction of outgrowths. Reproduced with permission from ref 38. Copyright 2014 Springer Nature.

### 2.2 Spatially Controlled Embedding of Nanoparticles (Ref 37)

ECL was used spatially controlled embedding of nanoparticles. This innovative technique enables the precise integration of nanoparticles into surfaces with nanometric resolution, offering a scalable solution for organizing nano-objects within thin oxide layers (Figure 2b). We demonstrated spatially controlled embedding using CoFe_2_O_4_ nanoparticles (NPs) as a model system.^37^ Initially, NPs are dispersed on a silicon surface. Subsequent application of ECL in an oxidative configuration leads to forming a SiO_2_ film that encapsulates the NPs. The quantity of embedded NPs is controlled by adjusting the concentration of the deposition solution. Importantly, NPs preserve their compositional and functional properties during the process. Figure 2b shows a pattern of parallel lines containing CoFe_2_O_4_ NPs.

### 2.3 Selective Electrochemical Decomposition of Outgrowths in Thin Films (Ref 38)

Beyond silicon-based applications, ECL has proven effective in selectively decomposing outgrowths in inorganic thin films (Figure 2c). These outgrowths (i.e., large grains typically exceeding 10 nm) often arise during deposition techniques such as laser ablation or pulsed electron ablation^39^ and can compromise device performance by causing short circuits or introducing defects in vertical junctions.^40^ We demonstrated that ECL can selectively decompose these outgrowths in conductive oxide thin films, making them insulating, without altering the underlying material properties.^38^ This was validated using La_0_._7_Sr_0_._3_MnO_3_ (LSMO), a benchmark material in spintronics and solid-state memory devices,^41^ known for its sensitivity to chemical composition. LSMO can be electrochemically modified via anodic or cathodic polarization, enabling targeted oxidation or reduction.

## 3 New Functionalities Enabled by Electrochemical Lithography

Beyond its traditional role in nanofabrication and patterning, we envisioned ECL as a powerful tool for creating novel functional properties with significant implications for advanced technologies, including memory devices and catalysis.

### 3.1 Regenerable Resistive Switching in Silicon Oxide-Based Nanojunctions

A particularly intriguing use of ECL is in resistive switching (RS), in which ECL provides unique advantages, such as regenerability, which can solve one of the most important problems regarding the durability of this type of device.^42,43^

Although electrochemically grown SiO_2_ is generally more porous and considered lower in quality compared to thermally grown oxide used in microelectronics, this lower quality can be advantageous in specific applications such as memristors, where it facilitates the RS by the formation of conductive Si filaments under voltage switching.^44,45^ RS in insulating or semiconducting materials has garnered significant attention due to the versatility and simple architecture of these devices, known as “memristors”. In its simplest form, a memristor consists of a thin film of insulating or semiconducting material sandwiched between two electrodes, with RS occurring upon the application of a high pulsed electric field, referred to as the formation step. Despite the recent progress, RS still face challenges, such as a limited number of program-erase cycles compared to traditional information storage devices and cross-talk issues through the thin film and sneak paths at cross-points.^46^ ECL has proven to be an effective method for producing RS Si/SiO_2_/Metal junctions (Figure 3a).^47^ The ability to generate SiO_2_ *in situ* using the same configuration of the device offers a key advantage: in cases of junction degradation, the active layer can be regenerated *in situ* by applying the same conditions used for silicon oxidation. Figure 3b shows the typical electrical behavior of the Si/e-SiO_2_/Metal junction, where SiO_*x*_ refers to electrochemically oxidaized silicon.

**Figure 3:**
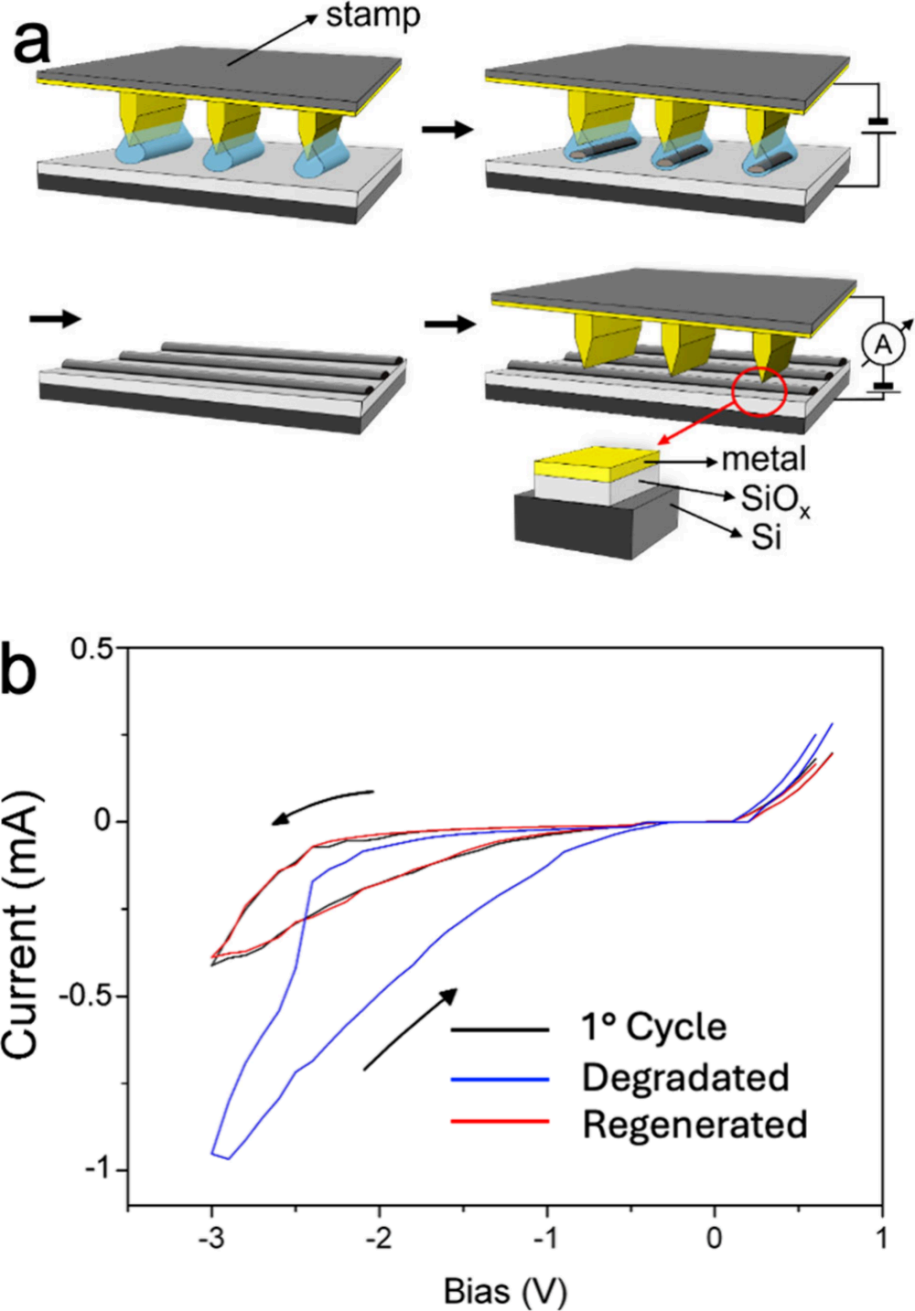
(a) Schematic diagram of device fabrication. (b) Electrical characterization of Si/e-SiO_2_/Metal memristor. The black curve shows the hysteretic I–V behavior following the forming step utilized for device activation. The blue curve illustrates a case of a degraded device after 10000 cycles. The red curve depicts the regenerated device, achieved by applying a +15 V pulsed bias that reoxidizes the semipermanent silicon filaments, resulting in device degradation. Reproduced with permission from ref 47. Copyright Wiley 2012.

The I–V curve of the device exhibits diode-like rectifying behavior.^48^ After a forming step that involves applying a large negative bias (typically −10 V), the I–V curve shows significant hysteresis and a sharp resistive switch at a negative bias. The device operates as a bipolar memristor, switching to a low-resistance state (ON) with a negative bias and to a high-resistance state (OFF) with a positive bias. The red curve in Figure 3b illustrates an example of device regeneration. The observed behavior and formation of outgrowths after several write/erase cycles indicate that resistive switching is driven by an electro-reductive/electromechanical process, leading to the formation of silicon nanoclusters following the breakdown of SiO_2_. These filaments are oxidized and destroyed when a positive bias is applied.^44,45^ ECL-based memristors offer several technological advantages over conventional devices: i- the ability to regenerate or repair the junction with an appropriate voltage cycle; ii- spatially controlled patterning of the insulating layer, fabricated in situ, which minimizes cross-talk issues through the thin film.

### 3.2 Defect Engineering: Nanopatterning of Atomic Sulfur Vacancy in MoS_2_ Surfaces

One exciting application is defect engineering, where ECL provides a powerful and precise way for introducing and controlling defects in materials, unlocking new functionalities and performance improvements. Defects such as heteroatoms and atomic vacancies are inherent in most materials acting as dopants, determining their chemical and physical properties.^49,50^ Although traditionally regarded as detrimental, defects can also be utilized to enhance or even enable new functionalities. Our recent studies have emphasized their potential to improve performance across areas from electronic transport to catalytic activity.

Our research has shown that ECL allows for precise creation and spatial control of reactive defect regions, interconnected by pristine­(unaffected) material zones. This patterning maintains the material’s inherent properties between treated areas, similar to an electrical circuit where conductive paths connect functional nodes. Such spatial control reduces the adverse effects of high defect concentrations on electrical conductivity, a key issue in defect engineering, and enables fine-tuning of functional properties by adjusting the local defect density.

The following section explores these capabilities in greater detail, illustrating how ECL can be leveraged to engineer defects with high precision and unlock new material functionalities such as magnetism and catalysis.

We recently employed ECL to create and accurately control high-density atomic sulfur vacancies (Vs) on the surface of a transition metal dichalcogenide thin film, specifically MoS_2_. This process enabled us to tune the material’s electronic properties and achieve specific functionalities in a controlled manner. Applying ECL in oxidative configuration, it oxidizes the material beneath the stamp protrusions, forming MoO_3_ structures that can be etched by water, producing a topographic pattern.^51,52^ Conversely, when ECL is applied in a reductive configuration, it leads to an electrochemical desulfurization of the MoS_2_ without significantly altering the morphology of the surface. The desulfurization of MoS_2_ occurs via a proton and electron transfer involving a sulfur atom of the surface, developing H_2_S gas that leaves behind Vs.^13,53^

$$S\mathrm{-Mo-}S^{*}+H^{+}+e^{-}\to S\mathrm{-Mo-}S^{*}\mathrm{‐‐‐}H,S^{*}=\mathrm{Surface}\;\mathrm{atom}\;\mathrm{of}\;{\mathrm{MoS}}_{2}$$ 1

$$S\mathrm{-Mo-}S^{*}\mathrm{-H}+H^{+}+e^{-}\to S\mathrm{-Mo-}V_{S}+H_{2}S_{\left(g\right)},V_{S}=\mathrm{Sulfur}\;\mathrm{vacancy}$$ 2

Figure 4 shows a SEM image of patterned areas that, importantly while in AFM investigation, do not show any significant morphological features (Figure 4a inset), when observed by SEM with decelerated electrons (<1 keV), the images display a distinct contrast in patterned samples that replicate the stamp features (Figure 4b). Notably, these evidence indicate that the process occurs exclusively at the surface and the observed contrast is compositional.

**Figure 4:**
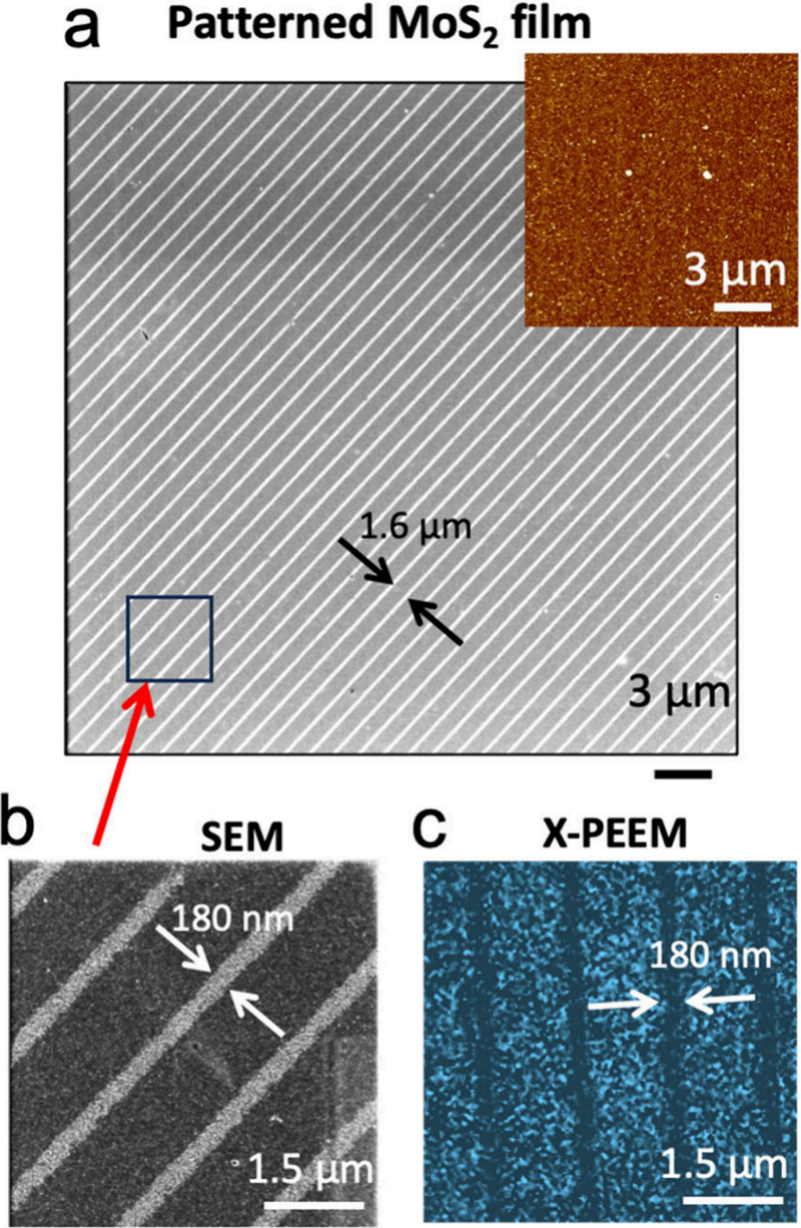
Characterization of patterned MoS_2_ thin films. (a) SEM image of ECL-treated MoS_2_ film acquired using decelerated electrons. The inset shows the corresponding AFM topography. (b) Detail of (a). (c) Energy-filtered X-PEEM image obtained collecting S 2p intensity (BE ∼ 163 eV). The contrast indicates local desulfurisation. Reproduced with permission from ref 13. Copyright Wiley 2025.

The local desulfurization in the printed zones is verified by X-ray photoemission electron microscopy (X-PEEM, Figure 4c), a surface-sensitive technique that offers both chemical sensitivity and high lateral resolution, as well as Raman spectroscopy investigations.^13^ Crucially, Vs introduce several new functionalities in these materials.^54^

### 3.3 Electrochemical Tuning of Energy Levels and Functional Properties in TMDs

Modifying surface chemistry and creating defects are fundamental strategies in material science, serving to tune their energy levels and functional properties. ECL has emerged as a valuable technique for adjusting electronic and functional characteristics, further enhancing the ability to tailor materials for specific applications. In particular, we utilized ECL to create patterns of sulfur atomic vacancies (Vs) in specific regions of a surface. The electronic properties of MoS_2_ with sulfur vacancies were tuned in two ways:

1. *Managing Local Defect Density*. The ECL process is additive, enabling control of local defect density by adjusting process time or repeating it in the same area. Importantly, their potential interaction significantly influences the effect on electronic levels and functional properties.
2. *Adjusting the Patterned Area*. The total number of fabricated defects can be regulated acting on the patterned area.

Kelvin Probe Force Microscopy (KPFM) and μ-differential reflectance spectroscopy (μ-DR) provide direct information on the ECL effects on electronic levels. Figures 5a and 5b show the topography and the corresponding surface potential image of a patterned sample. While the topography remains almost unaltered, the KPFM image reveals a high contrast in the patterned zones, reproducing the stamp motifs. The increase of the surface potential denotes a reduction in work function, ascribed to the introduction of Vs electronic midgap states near the conduction band edge. This behavior aligns with theoretical predictions and previous experimental KPFM studies.^55^

**Figure 5:**
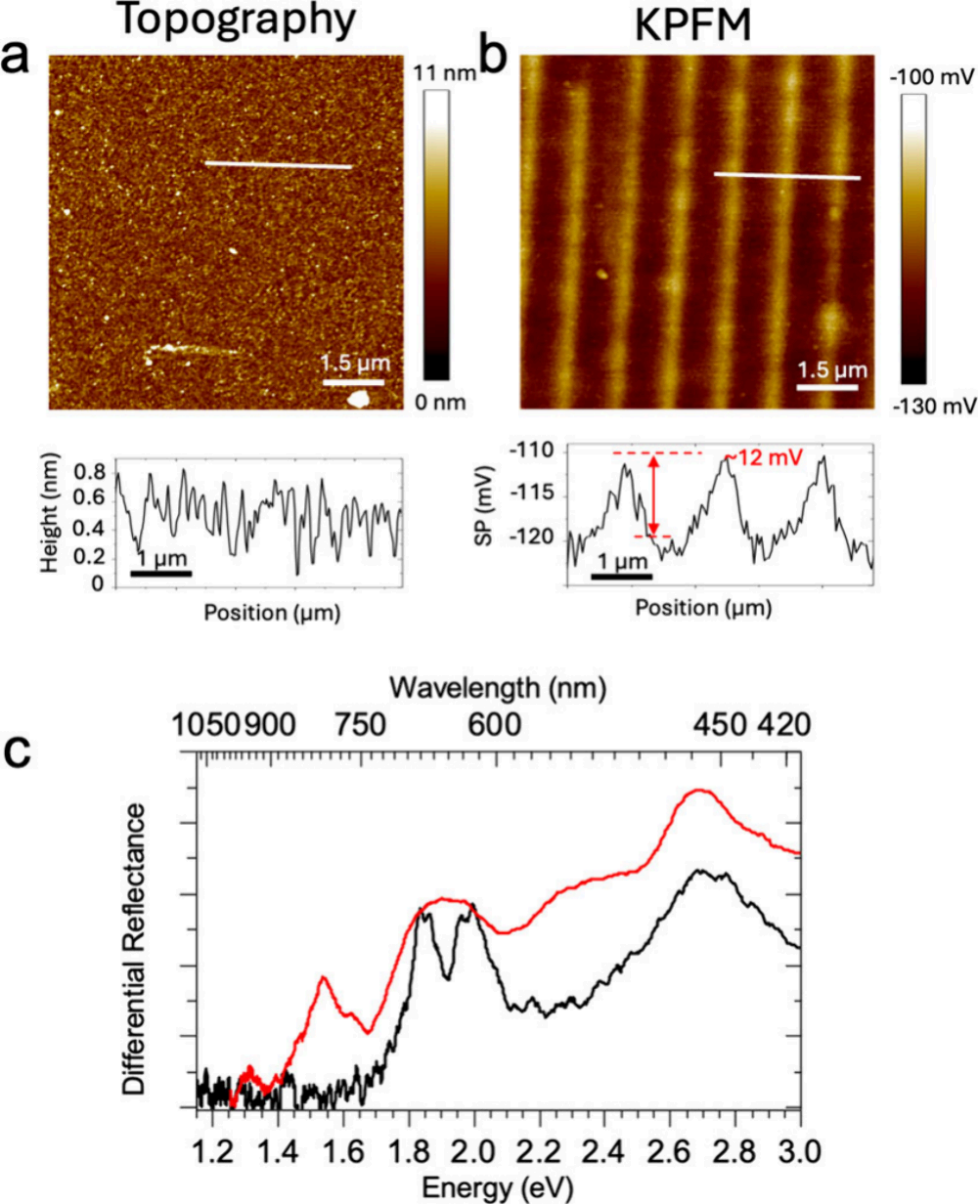
Topographic, electrical, and differential reflectance characterization of the patterned thin film. (a) AFM topographic image. (b) Corresponding KPFM map. The higher surface potential in correspondence with printed stripes suggests the presence of Vs resulting from the process. (c) Differential reflectance spectra of pristine MoS_2_ films (Black curve) and the patterned film. Bands (Red curve). Reproduced with permission from ref 13. Copyright Wiley 2025.

Figure 5c illustrates the μ-DR spectrum for defect-free crystalline MoS_2_ flakes, revealing distinct bands ″A″ (1.85 eV) and ″B″ (1.99 eV), in addition to a broad band ″C″ centered at 2.70 eV, associated with the thickness of the flakes. Conversely, the patterned film presents a clear band at 1.54 eV, which indicates the formation of midgap, unoccupied states. This observation offers direct experimental confirmation of localized, unoccupied in-gap states due to ECL desulfurization, aligning with theoretical and experimental observations.^56^

### 3.4 Evaluating the Interplay between Patterning and Functionality: Electrocatalysis in MoS_2_

To investigate the interplay between nanoscale patterning and functional performance, we examined the electrocatalytic behavior of MoS_2_ thin films, focusing on their activity toward the hydrogen evolution reaction (HER) in acidic media. Although MoS_2_, particularly in low-dimensional forms such as thin films and 2D flakes, where the basal plane is largely inert, is not considered an ideal catalytic system, it exhibits high sensitivity to defects.^14^ Indeed, defect-rich MoS_2_ structures are frequently proposed as promising noble-metal-free electrocatalysts for HER.^57^

While patterned samples may not consistently outperform uniformly treated films in terms of overall catalytic efficiency, nanoscale patterning plays a critical role in ensuring efficient electrical connectivity between active regions. Our group has demonstrated that vacancy sites (Vs) generated via electrochemical lithography (ECL) are electrocatalytically active and can be precisely controlled in terms of position, area, and density.^14^ This capability allows for the fine-tuning of catalytic activity, achieving performance levels comparable to leading 2D HER catalysts. Furthermore, the ability to spatially localize and modulate defect density via ECL presents a valuable strategy for integrating transition metal dichalcogenides (TMDs) into functional devices where both performance and spatial selectivity are essential.

We assessed the impact of nanopatterned, chemically active Vs on HER in acidic media by analyzing samples with varying geometries and patterned surface areas (0%, 10%, 20%, and 40%). Patterns were created through multiple ECL applications on the same region to (i) demonstrate the repeatability of desulfurization, (ii) locally tailor defect density, and (iii) explore the influence of geometry and patterned area on HER performance.

As shown in Figure 6, Linear Sweep Voltammetry (LSV) curves reveal that treated films exhibit significantly higher current densities (J) compared to untreated samples.

**Figure 6:**
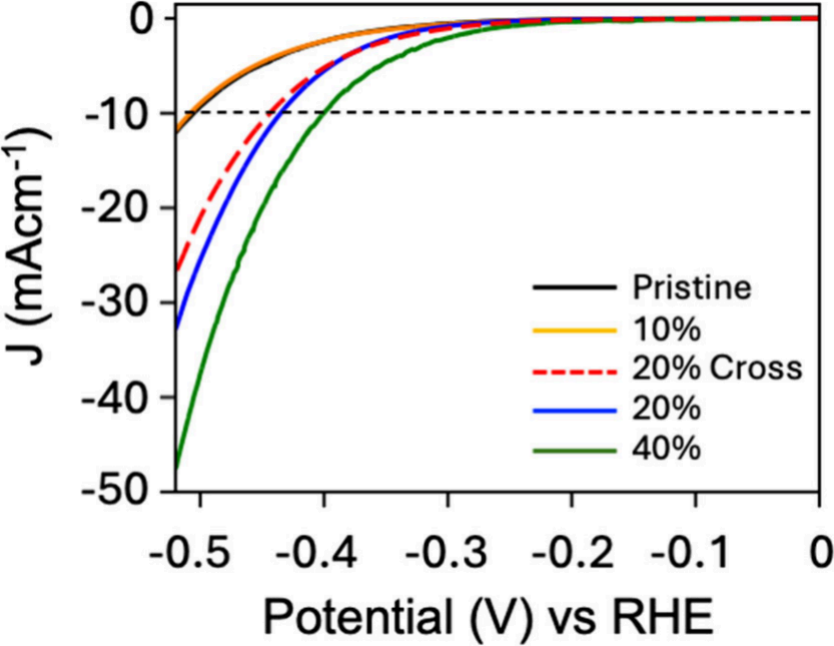
Catalytic characterization. LSV measurements of Mos_2_ films controlling the patterned area. Samples with a 20% treated area were produced by double printing the parallel lines (blue curve) and applying twice, rotating the stamp 90° between the two applications (red dashed curve). Reproduced with permission from ref 13. Copyright Wiley 2025.

The electrocatalytic activity of pristine MoS_2_ arises from intrinsic defects within the film. The LSV curves show a clear correlation between catalytic activity and the extent of the patterned area, confirming that ECL generates catalytically active sites in proportion to the treated surface area. Notably, samples with 40% patterned area demonstrate high electrocatalytic performance, achieving an overpotential of −0.40 V at a current density of 10 mA cm^–2^. In contrast, samples with only 10% treated area show limited activity, indicating that substantial enhancement in electrochemical performance occurs only when the patterned area exceeds a critical threshold. This behavior is attributed to surface outgrowths that act as spacers, hindering the formation of nanoelectrochemical cells around these features and effectively reducing the active area. When comparing LSV curves across samples with different geometries, no significant differences were observed. Although ECL-treated MoS_2_ films exhibit electrocatalytic activity more than five times greater than pristine samples, their overall performance remains limited compared to conventional MoS_2_ electrodes. This limitation is primarily due to the nearly flat morphology of our samples, which results in a surface area significantly smaller than that of the porous architectures typically employed in catalytic applications.

### 3.5 Molecular Assisted Electrochemical Atomic-Scale Patterning

Recent breakthroughs in materials science, particularly in the field of defect engineering, are driving a paradigm shift in patterning strategies, transitioning from the nanoscale toward atomic-scale precision. This shift is motivated by the profound influence that atomic defects, such as vacancies and substitutional atoms, exert on the properties of materials. The nature and spatial distribution of these defects at the atomic level critically determine material performance and stability, underpinning their success in a wide range of technological applications. Among the various types of defects, isolated atomic defects are especially significant as they are the most stable and capable of imparting a remarkable array of functionalities. However, the ability to pattern single-atom defects with atomic precision while maintaining scalability remains a major challenge. Current state-of-the-art techniques, such as SPM, offer atomic-scale resolution but suffer from low throughput and require complex instrumentation and stringent experimental conditions.

To address these limitations, we suggest a new approach that uses molecular systems as tools for electrochemical patterning. These molecules serve as nanoscale templates, allowing for single-atom precision over large areas—an achievement not possible with current parallel electrochemical or SPM-based methods.

In pursuit of this goal, we recently introduced an innovative approach in which specific molecular species effectively replace the SPM probe. This idea is inspired by the behavior of certain coordination compounds, such as phthalocyanines and porphyrins, which can template, rearrange, or exchange atoms with a surface in a way that resembles atom manipulation via SPM. Figure 7a schematically represents this process.

**Figure 7:**
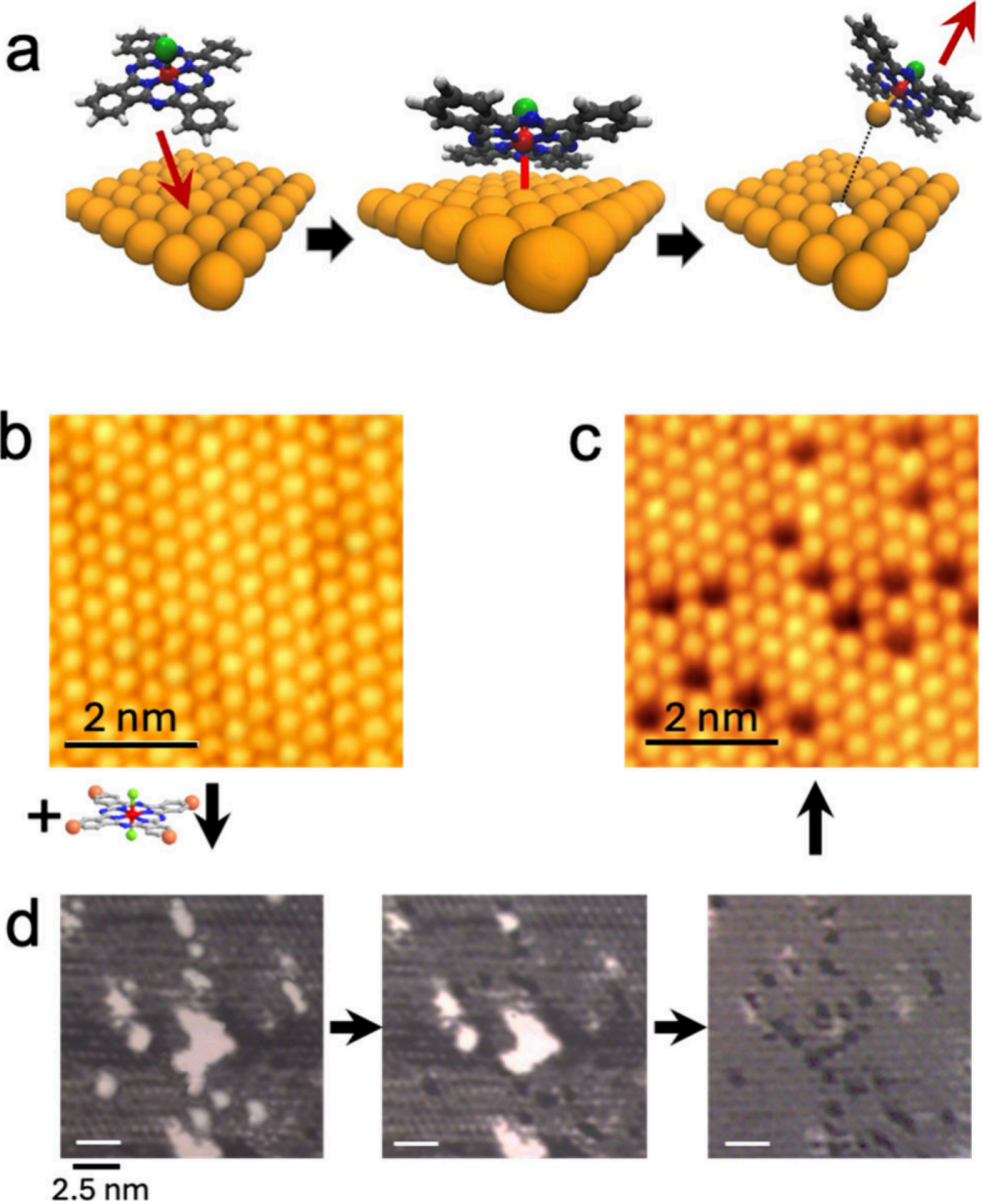
Concept of molecular assisted single atom desorption. (a) A coordination compound is deposited on a surface. It bonds with an atom of the surface through its metal and picks it up, causing molecule desorption along with the extracted atom. (b) STM images illustrate pristine surfaces (bar = 2 nm). (c) Corresponding image at the end of the process. (d) Time-lapse of electrochemical desorption related to the formation of *I* atomic vacancies (dark spots) in relation to the desorbed molecule (bar = 2.5 nm). Reproduced with permission from ref 58. Copyright Wiley 2021.

Briefly, a molecule of a coordination compound is deposited on a substrate, bonding to a specific atom on the surface through surface atom complexation. Then, upon electrochemical action, it is desorbed, taking the bonded atom with it and creating an atomic vacancy at a precise location on the surface. Utilizing thin films of coordination compounds instead of an SPM probe addresses the primary limitations of SPL, as it operates as a parallel process with all adsorbed molecules functioning simultaneously and has no spatial restrictions.

Molecular-assisted electrochemical single-atom desorption was demonstrated on both halogen- and dichalcogenide-terminated surfaces. Figure 7b illustrates an example applied to iodine-modified Ag surfaces, a particularly stable system recognized for its high crystallinity, room-temperature stability, and manipulability in air, alongside layers of sulfur and selenium.

We are aware that further efforts are needed to direct ″swarms″ of molecular complexants to specific atoms on the surface, facilitating long-distance positional control of molecules and the formation of vacancies in predetermined patterns. In this regard, the exceptional capability of coordination compounds, such as phthalocyanines and porphyrins, to create self-assembled monolayers, which can be geometrically controlled through chemical design, may provide substantial support.

## 4 Perspectives of Stamp-Assisted Electrochemical Lithography

ECL has demonstrated itself to be a reliable and adaptable technique, and its application is anticipated to extend to a broad range of materials. Among the most compelling opportunities is the spatially controlled engineering of defects. This capability could provide a breakthrough in defect engineering by enabling the creation of highly organized, well-defined, and spatially localized defects, such as single atomic vacancies, within regions that retain the pristine properties of the original material. This degree of control is vital for advancing next-generation electronic, photonic, and quantum devices. Looking ahead, a key objective is to push the spatial resolution of ECL from the nanoscale toward the atomic scale. This ambitious goal may be achieved by integrating conventional ECL with Molecular-Assisted Electrochemical Atomic-Scale Patterning, a hybrid approach that could unlock unprecedented precision in material modification. Such advancements would position stamp-assisted ECL as a transformative tool for atomic-scale fabrication, opening new frontiers in nanotechnology and materials science.

While current implementations depend on custom-built instrumentation, future efforts will concentrate on developing more refined and accessible apparatus. Although important for broader adoption, these technological improvements are secondary to the scientific potential of the method, which remains the primary driver of ongoing research.

## 5 Concluding Remarks

This Account delves into the groundbreaking technique of surface electrochemical nanopatterning, emphasizing its remarkable potential as a robust fabrication tool for both morphological and chemical patterning. ECL facilitates the precise tuning of electronic and functional properties across various materials. Our findings illustrate how electrochemical processes within confined environments not only enhance existing properties but also unlock exciting new functionalities in materials, including the *in situ* synthesis and nanoembedding of nanoclusters and nanoparticles.

By utilizing electrochemical nanopatterning in confined spaces, we achieve meticulous modifications of materials within spatially defined zones. This cutting-edge advancement enables the creation of small, regularly spaced, addressable, and highly functional nanostructures. Such capabilities lead to significant enhancements in material properties, effectively addressing critical challenges, such as the localized reduction of electrical conductivity often associated with defects in transition metal dichalcogenides. In many instances, this approach allows for both the control and generation of entirely new functionalities, particularly in the realms of magnetic and catalytic properties. These enhanced attributes have far-reaching implications for technological advancements, amplifying device performance and expanding the potential applications of advanced materials.

The stamp-assisted process, designed for large-area applications, opens up new possibilities for technological advancements based on the extraordinary phenomena and properties revealed by scanning probe nanolithography over the past 25 years, which have historically been limited by throughput constraints. Moreover, electrochemical nanolithography establishes a foundation for several bottom-up nanofabrication techniques, offering direct benefits across diverse application domains. Looking ahead, although challenges remain in utilizing confined electrochemical nanopatterning for emerging fields such as atomic-scale manipulation, its potential is positioned to significantly influence technological applications in both established and innovative devices.
